# Properties of the phage-shock-protein (Psp) regulatory complex that govern signal transduction and induction of the Psp response in *Escherichia coli*

**DOI:** 10.1099/mic.0.040055-0

**Published:** 2010-10

**Authors:** Goran Jovanovic, Christoph Engl, Antony J. Mayhew, Patricia C. Burrows, Martin Buck

**Affiliations:** Division of Biology, Sir Alexander Fleming Building, Imperial College London, South Kensington Campus, London SW7 2AZ, UK

## Abstract

The phage-shock-protein (Psp) response maintains the proton-motive force (pmf) under extracytoplasmic stress conditions that impair the inner membrane (IM) in bacterial cells. In *Escherichia coli* transcription of the *pspABCDE* and *pspG* genes requires activation of *σ*^54^-RNA polymerase by the enhancer-binding protein PspF. A regulatory network comprising PspF–A–C–B–ArcB controls *psp* expression. One key regulatory point is the negative control of PspF imposed by its binding to PspA. It has been proposed that under stress conditions, the IM-bound sensors PspB and PspC receive and transduce the signal(s) to PspA via protein–protein interactions, resulting in the release of the PspA–PspF inhibitory complex and the consequent induction of *psp*. In this work we demonstrate that PspB self-associates and interacts with PspC via putative IM regions. We present evidence suggesting that PspC has two topologies and that conserved residue G48 and the putative leucine zipper motif are determinants required for PspA interaction and signal transduction upon stress. We also establish that PspC directly interacts with the effector PspG, and show that PspG self-associates. These results are discussed in the context of formation and function of the Psp regulatory complex.

## INTRODUCTION

The phage-shock-protein (Psp) response maintains the proton-motive force (pmf) under extracytoplasmic stress conditions (e.g. upon secretin pIV production) that impair the integrity of the inner membrane (IM) and dissipate the pmf (reviewed by Darwin, 2005; see also Jovanovic *et al.*, 2006). This adaptation to stress has been shown to be important for growth and virulence of some enterobacterial pathogens (reviewed by Darwin, 2005, 2007; Rowley *et al.*, 2006). Transcription of the *psp* genes is controlled by *σ*^54^-RNA polymerase and activated by the bacterial enhancer-binding protein PspF (reviewed by Model *et al.*, 1997; Darwin, 2005; Wigneshweraraj *et al.*, 2008). In *Escherichia coli* the PspF regulon consists of the *pspABCDE* operon, the adjacent *pspF* gene and the *pspG* gene (reviewed by Model *et al.*, 1997; Darwin, 2005). In many other enterobacteria, the *pspF* and *pspABC* genes are highly conserved (Huvet *et al.*, 2009). Under non-stress growth conditions, PspA inhibits the ATPase activity of PspF and negatively controls expression of the *psp* genes (Elderkin *et al.*, 2002, 2005; Joly *et al.*, 2009). It is proposed that under many *psp*-inducing stress conditions, the IM-bound sensors PspB and PspC act as positive regulators that receive and transduce the stress signal(s) to PspA via protein–protein interactions (Adams *et al.*, 2003; Maxson & Darwin, 2006; Jovanovic *et al.*, 2006, 2009). This ultimately results in the release of the PspA–PspF inhibitory complex, leading to *psp* induction and greatly elevated expression of the Psp proteins.

Recently, we provided evidence that during microaerobic growth the ArcAB system contributes to a Psp signal-transduction pathway in a PspBC-dependent manner and proposed that at least two signals could be recognized by PspBC for full pIV-dependent *psp* induction – caused by the mislocalization of pIV in the IM (Jovanovic *et al.*, 2009). These data suggested that a double check-point for PspBC-specific *psp* induction could exist, potentially explaining the requirement for two sensor proteins. Importantly, in the absence of stress, PspBC overexpression has been shown to strongly induce *psp* (Weiner *et al.*, 1991; Maxson & Darwin, 2006), implying that under these conditions PspBC may bypass upstream signalling pathways and directly release the PspA–PspF inhibitory complex. However, the mechanistic basis of this effect is unknown. In light of these findings a Psp[F–A–C–B]–ArcB regulatory complex (under non-stress conditions) was proposed (Jovanovic *et al.*, 2009), but importantly only pairwise interactions between PspA–PspF, PspA–PspC, PspB–PspC and PspB–ArcB have been identified (Elderkin *et al.*, 2002; Adams *et al.*, 2003; Elderkin *et al.*, 2005; Maxson & Darwin, 2006; Jovanovic *et al.*, 2009). PspG is an IM effector protein not required for *psp* induction (Lloyd *et al.*, 2004), but it may co-localize with PspA (Engl *et al.*, 2009). Interestingly, it was observed that overexpression of PspG upregulates genes involved in microaerobic and anaerobic respiration (most of them ArcAB-regulated) (Jovanovic *et al.*, 2006). The role of PspG and its potential association with the Psp regulatory complex has yet to be established.

In this work we dissect further the Psp regulatory complex in terms of protein–protein interactions and define the functional determinants in PspB and PspC that are required for pIV-dependent *psp* induction.

## METHODS

### Bacterial strains.

The bacterial strains used in this study are listed in Supplementary Table S1, available with the online version of this paper. Strains were constructed by transduction using the P1*_vir_* bacteriophage (Miller, 1992).

### Media and growth conditions.

All strains were routinely grown under microaerobic conditions in Luria–Bertani (LB) broth or on LB agar plates at 37 °C (Miller, 1992). For microaerobic growth, overnight cultures of cells were diluted 100-fold (OD_600_ ∼0.025) and shaken at 100 r.p.m. For the bacterial two-hybrid (BACTH) assays, strains were grown in LB at 30 °C. For single-molecule fluorescence imaging of PspG–GFP, cells were grown in minimal medium as described previously (Engl *et al.*, 2009). Induction of the pBAD *ara* promoter was achieved with either 0.001 % or 0.02 % (final – as indicated) l-arabinose (Ara). Antibiotics were used at the following concentrations: ampicillin, 100 μg ml^−1^; kanamycin, 25 or 50 μg ml^−1^, as indicated; chloramphenicol, 30 μg ml^−1^; and tetracycline, 10 μg ml^−1^.

### DNA manipulations.

Plasmids used in this study are listed in Supplementary Table S1. All constructs were verified by DNA sequencing, and protein production was verified using Western blotting. By site-directed mutagenesis (QuikChange, Agilent Technologies) using the appropriate primers and either pAJM2 (encodes wild-type [WT] PspC) or pAJM3 (encodes WT PspBC) as a template, we constructed pAJM6–8, pAJM11–13, pGJ54, pGJ55 and pGJ57. Using either pAJM8, pAJM13 or pAJM11 as a template, we constructed pAJM5, pAJM10, pGJ60 and pGJ61. The BACTH fusion proteins were created by fusing (i) the N-terminus of the protein of interest to either pKT25 or pUT18C or (ii) the C-terminus to either pKNT25 or pUT18 (Table 1). Genes were amplified using primers that introduce either *Xba*I-(no stop codon)-*Kpn*I and *Hin*dIII-(no stop codon)-*Kpn*I or *Xba*I-(stop codon)-*Kpn*I restriction sites.

### *In vivo* bacterial BACTH system.

**Fig. 1.**
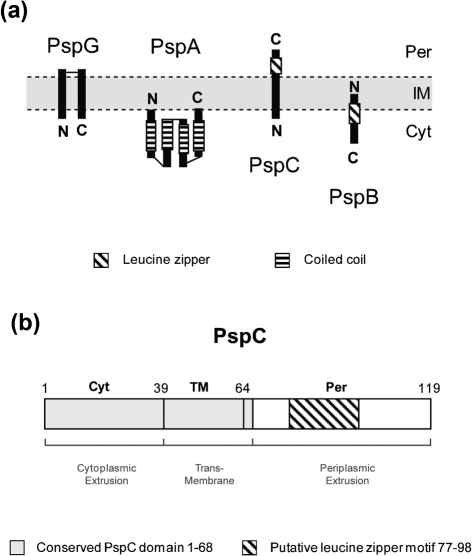
Topologies of Psp proteins. (a) Schematic representation of PspA, PspB, PspC and PspG topologies according to Elderkin *et al.* (2005), Jones *et al.* (2003), Kleerebezem *et al.* (1996) and Engl *et al.* (2009), respectively. Per, periplasm; IM, inner membrane; Cyt, cytoplasm; N, N-terminus of the protein; C, C-terminus of the protein. (b) Representation of the predicted conserved domain organization of PspC: the cytoplasmic extrusion domain (Cyt, residues 1–39), the transmembrane portion (TM, residues 40–64), the periplasmic extrusion domain (Per, residues 65–119) and the conserved PspC domain (residues 1–68, light grey). The putative leucine zipper motif (LeuZ; residues 77–98) is as indicated. The numbering refers to *E. coli* PspC.

The Cya-based BACTH assay was used to study *in vivo* protein–protein interactions (Karimova *et al.*, 1998, 2005). Protein fusions (see Table 1 and Fig. 1) were assayed in BTH101 cells as described previously (Jovanovic *et al.*, 2009). Interactions were quantified by measuring *β*-galactosidase activity in liquid cultures (see below). Chromosomal LacZ expression threefold above the negative control (vector alone) value was scored as a positive interaction signal.

To test protein–protein interactions in the absence of natively produced Psp proteins that may provide or facilitate binding interactions by a bridging effect, we constructed a derivative of the BTH101 strain carrying the Δ*pspF* mutation – thereby preventing expression of the native Psp proteins (see Supplementary Table S2). The wild-type *pspF* gene was replaced by Δ*pspF* : : Kan, yielding MVA99 (see Supplementary Table S1). The Kan cassette from Δ*pspF* : : Kan was eliminated and the marker-less *pspF99* variant (strain MVA100) constructed using plasmid pCP20 and the method described by Cherepanov & Wackernagel (1995) (see Supplementary Table S1). The strain was verified by PCR.

### *β*-Galactosidase (*β*-Gal) assays.

Activity from a chromosomal Φ(*pspA*–*lacZ*) transcriptional fusion was assayed to gauge the level of *psp* expression, while activity of the native chromosomal *lacZ* was measured in the BACTH assays. Overnight cultures grown at 37 °C (or 30 °C for the BACTH assays) were diluted 100-fold and grown under the same conditions until mid-exponential phase. Cultures were induced (with Ara or 0.5 mM IPTG for BACTH, as indicated) for 1 h and then assayed for *β*-Gal activity (Miller, 1992). For all *β*-Gal assays, mean values from six samples taken from technical duplicates of three independently grown cultures of each strain were used to calculate activity. The data shown in the figures are the mean values with sd error bars.

### Bacterial cell fractionation.

Bacterial cultures were separated at mid-exponential phase into soluble and membrane fractions by a lysozyme-EDTA-osmotic shock protocol and the inner and outer membranes were selectively extracted with Triton X-100 (Russel & Kaźmierczak, 1993). Samples were analysed by Western blotting.

### Western blotting.

Bacterial cells were harvested at mid-exponential phase and resuspended in a mix of 30 μl 4 % SDS and 30 μl Laemmli buffer (Sigma). Samples were normalized according to cell growth measured as OD_600_, separated by SDS-PAGE and transferred onto PVDF membrane using a semidry transblot system (Bio-Rad). Western blotting was performed as described by Elderkin *et al.* (2002) using antibodies to PspA (1 : 10 000) (Jones *et al.*, 2003), PspB (1 : 5000), PspC (1 : 5000), PspG (1 : 1000) (Jovanovic *et al.*, 2006) or pIV (1 : 10 000). Proteins were detected using the ECL plus Western Blotting Detection kit (GE Healthcare). Images were captured in a FujiFilm Intelligent Dark Box by an image analyser with a charge-coupled-device camera (LAS-3000). Densitometry analysis was performed with MultiGauge 3.0 software (FujiFilm USA) and quantification (results expressed in arbitrary units) was performed using the aida software.

### Confocal fluorescence microscopy to assess membrane electron potential (Δ*ψ*).

The Δ*ψ* component of pmf was measured using the JC-1 dye method (Becker *et al.*, 2005) as described previously (Jovanovic *et al.*, 2006; Engl *et al.*, 2009). The green to red fluorescence emission ratio (530/590 nm) was calculated from 100 individual cells taken from technical duplicates of three independently grown cultures of each strain. The threshold to distinguish pmf differences was chosen based on the Δ*ψ* (equivalent to pmf under given experimental conditions) of the WT strain under non-stress growth conditions.

### Single-molecule fluorescence imaging of PspG *in vivo*.

Single molecules in fluorescent PspG–GFP complexes (MG1655 Δ*pspG*/PspG–GFP) were imaged *in vivo* (Engl *et al.*, 2009). PspG–GFP-expressing cells were mounted on a cover glass and images taken at 80 ms per frame. The fluorescent PspG–GFP complexes were analysed using the ImageJ software (http://rsb.info.nih.gov/ij/) (Abramoff *et al.*, 2004), with GFP from MG1655/pDSW209 cell lysates as a reference.

### Protein purification.

*E. coli* Top10 or Top10Δ*pspA* cells transformed with either pRD047 (encoding PspB and non-tagged PspC) or pRD047His (encoding PspB and His-tagged PspC) were grown in LB at 37 °C until they reached OD_600_ ∼0.5, at which point the culture was shifted to 30 °C. Overexpression was induced by addition of 0.02 % Ara and the cultures grown for a further 3 h. Cells were harvested at 8000 r.p.m. for 10 min at 4 °C; the pellet was resuspended in 20 ml buffer A (50 mM sodium phosphate pH 7, 300 mM NaCl, 5 %, v/v, glycerol) supplemented with Complete Protease Inhibitor (Roche) and lysed by sonication. The supernatant was loaded onto a 5 ml HiTrap metal-chelating column (GE Healthcare) pre-charged with NiCl_2_ and the protein eluted with buffer A supplemented with 1 M imidazole. Purified proteins were detected by Western blotting (see above).

### Bioinformatic methods.

Multiple sequence alignment was performed using Multalin software version 5.4.1 (http://bioinfo.genotoul.fr/multalin/) (Corpet, 1988). Protein domain analysis was performed using Pfam version 21.0 (http://www.sanger.ac.uk/pfam) (Finn *et al.*, 2006). Transmembrane (TM) helices prediction, localization and topologies were performed using tmhmm (http://www.cbs.dtu.dk/services/TMHMM/, V 2.0) (Kahsay *et al.*, 2005), hmmtop (http://www.enzim.hu/hmmtop/, V 2.0) (Tusnády & Simon, 2001), TopPred2 (http://mobyle.pasteur.fr/cgi-bin/portal.py?form=toppred) (von Heijne, 1992) or topcons (http://topcons.net/) (Bernsel *et al.*, 2009).

## RESULTS AND DISCUSSION

### PspG associates with Psp proteins known to form regulatory complexes

Previous studies suggested the presence of Psp regulatory complexes (Adams *et al.*, 2003; Maxson & Darwin, 2006; Jovanovic *et al.*, 2009). To determine whether PspG is able to interact with any of the proteins that constitute these regulatory complexes we performed pairwise BACTH interaction assays with PspA, PspB, PspC, ArcB and PspG (Table 1 and data not shown). The topologies of PspB, PspC and PspG (Fig. 1a) (reviewed by Darwin, 2005; see also Engl *et al.*, 2009) were taken into account when constructing and analysing the fusion proteins (e.g. fusions to protein regions embedded in the IM could interfere with its localization). As indicated in Table 1, we found that (i) PspA interacts with itself and PspC, (ii) PspB can interact with itself, PspC and ArcB, (iii) PspC can interact with PspA, PspB and PspG, (iv) PspG can interact with itself and PspC, and (v) ArcB interacts with PspB. The interactions obtained do not appear attributable to general non-specific IM protein interactions since the IM proteins PspB and PspC show distinctive sets of positive results (see Table 1). Any bridging effects of natively produced Psp proteins however cannot be discounted, unless BACTH assays are performed in a host strain unable to express Psp (e.g. Δ*pspF*; see below and Supplementary Table S2).

**Fig. 2.**
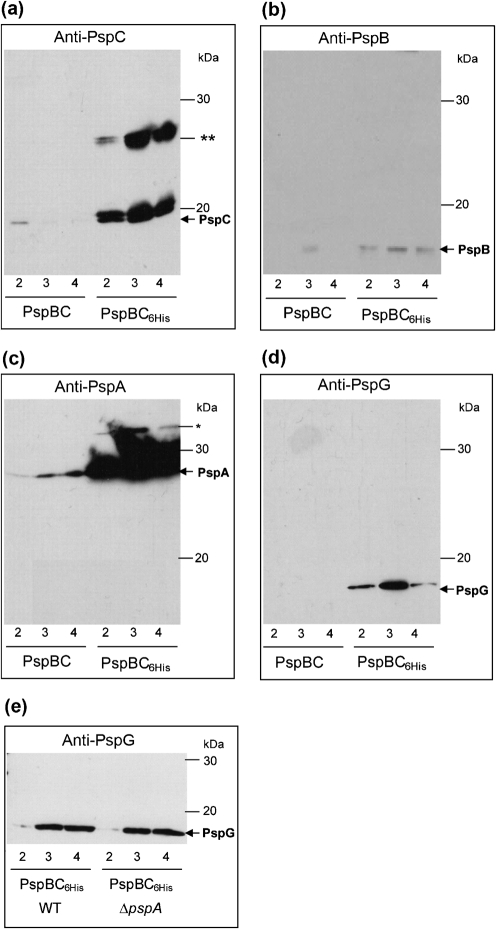
Co-purification of PspA, PspB and PspG with PspC_6His_. PspC_6His_ co-expressed with PspB (pRD047His) was purified using metal-affinity chromatography (see Methods). As a control non-tagged PspC co-expressed with PspB (pRD047) was also purified. Peak fractions from both purifications (corresponding to fractions 2–4), were visualized using Western blotting and (a) anti-PspC, (b) anti-PspB, (c) anti-PspA or (d) anti-PspG antibodies as indicated. In (a) the positions of monomeric PspC (PspC; arrow) and an additional slower-migrating anti-PspC-reactive (double asterisk) species are as indicated. PspB (b), PspA (and non-specific band, asterisk) (c) and PspG (d) co-purified with PspC_6His_ fractions (2–4) but not with PspC (non-tagged). (e) PspC_6His_ co-expressed with PspB was also purified from a Δ*pspA* strain and the peak fractions were visualized using Western blotting and anti-PspG antibodies. The positions of molecular mass marker proteins (kDa) are indicated.

In order to provide evidence that Psp proteins may indeed form a complex and to determine whether PspG is truly associated with PspC, we performed pull-down assays using PspC_6His_ co-expressed with WT PspB (since overexpression of PspC acts as a *psp* inducer, causing a drop in pmf – see below). We specifically selected PspC given that the pairwise interaction data suggest that PspC interacts with PspA, PspB and PspG (Table 1). We reasoned that PspC would, by means of protein–protein interactions, specifically pull down Psp proteins involved in the regulatory complex during protein purification (via the His-tag on PspC). As a control, we performed the chromatography assay with non-tagged PspC to demonstrate that any Psp proteins identified in the eluted fractions are present due to a specific interaction with PspC_6His_. When we analysed the PspC_6His_ elution fractions we found that PspA, PspB and PspG all co-purified with PspC_6His_ (Fig. 2a–d) – co-purification of PspA,B,C suggests that a Psp[A–C–B] regulatory complex exists, to which PspG may associate. PspG also co-purifies with PspC_6His_ when expressed in cells lacking PspA (Fig. 2e), further indicating that PspG interacts directly with PspC.

Potentially, co-expression of PspBC leads to an ‘active’ form of Psp(B)C that can specifically interact with PspA and possibly PspG. Such complexes may have significant implications in understanding the signalling cascade and activation of the Psp response as well as the effector function of PspG. Importantly, a reduced *psp* operon, *pspACG*, found in *Aeromonas* species (M. P. H. Stumpf, personal communication) further suggests that functional interactions occur between PspA, PspC and PspG.

**Fig. 3.**
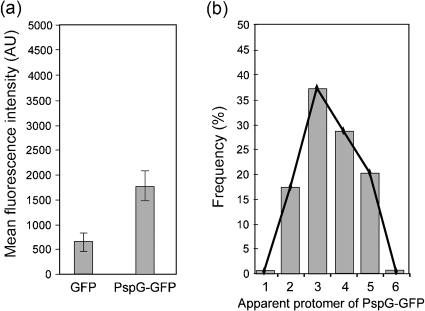
PspG–GFP forms higher oligomers *in vivo*. The number of molecules in fluorescent PspG–GFP complexes was estimated using ImageJ software. The intensity of a single pixel in a fluorescent PspG–GFP (pGJ7) cluster expressed in *E. coli* MG1655 Δ*pspG* cells (MVA40) was measured and compared to GFP fluorescence in *E. coli* MG1655 cell lysates harbouring pDSW209 (GFP alone). (a) The mean fluorescence intensity of 50 PspG–GFP complexes within living cells was on average at least three times higher than that of GFP spots, suggesting that PspG can form at least a dimer/trimer *in vivo*. (b) The frequency distribution among the 50 complexes analysed illustrates that PspG–GFP self-assembles into a single major distinct oligomeric class.

Given the ability of PspG to self-associate (as suggested in the BACTH assay; Table 1) and since the oligomeric state of PspG could be important for its effector function, we analysed PspG–GFP complexes in live MG1655Δ*pspG* cells using single-molecule fluorescence imaging and objective-type total internal reflection fluorescence (TIRF) (see Methods, Engl *et al.*, 2009 and e.g. Haggie & Verkman, 2008). The results illustrate that PspG–GFP can form complexes as large as pentamers/hexamers but the majority are dimers/trimers (Fig. 3).

Results from the BACTH assay also indicated that PspG, when expressed from the high-copy-number plasmid pUT18, interacts with ArcB (data not shown). However, when PspG was expressed from the low-copy-number plasmid pKNT25 no interaction with ArcB was detected (Table 1). As we were unable to detect an interaction between PspG and ArcB with the low-copy-number plasmid expressing PspG, we infer that the observed interaction either may be non-specific or may reflect the action of PspG as an effector when overproduced (Jovanovic *et al.*, 2006) – the later possibility has yet to be explored.

### PspC does not stably self-associate

**Fig. 4.**
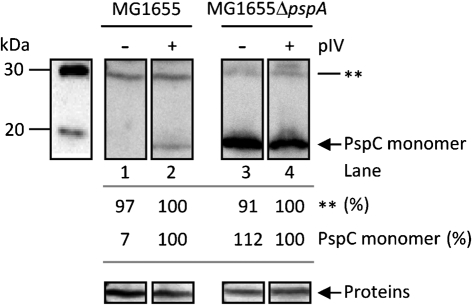
Oligomerization state of PspC. Western blots (using anti-PspC) illustrating PspC expression from the chromosome in either WT (MG1655) or Δ*pspA* (MG1655Δ*pspA*) cells (in the absence or presence of pIV). The positions of species that specifically cross-react with anti-PspC are highlighted as monomeric PspC (13.5 kDa; arrow) and a putative dimer (double asterisk). The positions of the marker proteins (kDa) are indicated. Below: the relative expression levels of monomeric PspC (labelled as PspC monomer) and the putative PspC dimer (double asterisk) were quantified within each strain tested, and the results expressed as a percentage of the induced corresponding protein band (+pIV; lanes 2 and 4, 100 %). ‘Proteins’ refers to the loading control. Importantly, these results demonstrate that in the absence of PspA (lanes 3 and 4), the relative expression levels of monomeric PspC are clearly highly elevated compared to WT in the presence of pIV, whereas the putative dimer expression level remains relatively unchanged, suggesting that this band corresponds to an unspecific anti-PspC cross-reacting species.

Peak fractions from the PspBC_6His_ purification were analysed by SDS-PAGE (Fig. 2a) and found to contain a number of PspC cross-reacting species (Supplementary Figs S1Ai, S2C, Dii, S3Bi and C) – one which migrates as a monomer and one as a putative dimer. It has been proposed that changes in pmf that occur upon extracytoplasmic stress may cause the stable self-association of PspC – reported by Adams *et al.* (2003) as an SDS-PAGE-stable dimeric form of PspC. Although we showed that co-expression of PspBC does not change pmf (see below), and BACTH analysis failed to detect pairwise PspC–PspC interactions in either *Yersinia enterocolitica* (Maxson & Darwin, 2006) or *E. coli* (Table 1), we cannot discount the possibility that either (i) the conformation of the fusion proteins is unfavourable for detecting PspC self-association or (ii) PspC may stably associate with an unknown protein to form the apparent ‘dimer’ species. Alternatively, PspC self-association may only occur upon pIV stress or when PspC is highly overproduced, since the apparent dimer was only observed when PspC was overproduced in a Δ*pspC* strain (Supplementary Fig. S3Bi and C). To investigate this further, and to address whether formation of a putative PspC dimer can be observed under physiological conditions, we analysed the expression of chromosomally encoded PspC in WT cells and cells lacking the negative regulator PspA (Δ*pspA*) in the presence and absence of pIV (Fig. 4). Fully consistent with its role in *psp* induction, pIV elevated Psp expression in WT cells (observed as an increase in monomeric PspC protein levels). Further, in cells lacking PspA, monomeric PspC expression remained high regardless of the presence of pIV (indicative of deregulated Psp expression). Notably, the intensity of the slower-migrating anti-PspC-reactive complex (the putative PspC dimer) remained unchanged under conditions where PspC was expressed at different levels (Fig. 4), suggesting that under physiological conditions PspC does not form a stable complex with either itself or PspA (since an identical anti-PspC-reactive complex, the putative PspC dimer, is observed upon overexpression of PspC in a Δ*pspA* strain). The lower level of PspC expression in cells encountering pIV stress versus cells lacking negative regulation (Fig. 4) is fully consistent with our findings that even under pIV stress conditions, PspA is still imposing apparent negative regulation (Engl *et al.*, 2009; Jovanovic *et al.*, 2009).

These results show that the apparent dimer species recognized by the PspC antibodies does not follow PspC expression levels, and therefore should not be considered as a stable PspC dimer. Taking these findings together with the BACTH assays, we conclude that under the physiological conditions tested PspC does not stably self-associate.

### PspB and PspC act together to directly release negative regulation of *psp*

**Fig. 5.**
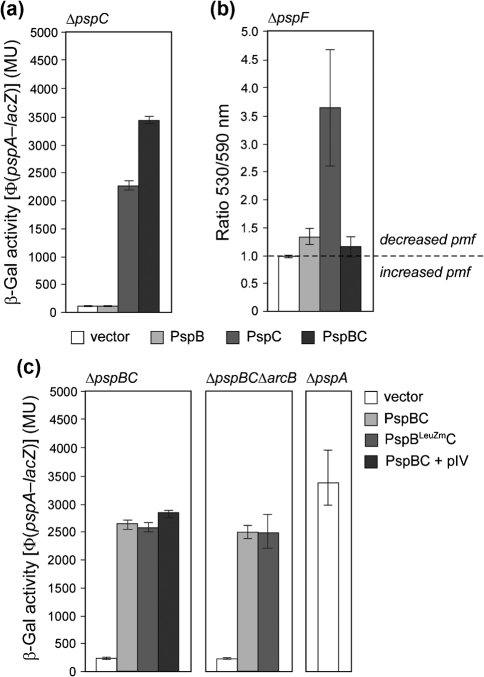
*psp* expression in the presence of overexpressed PspBC. (a) Induction of chromosomal Φ(*pspA–lacZ*) in a Δ*pspC* strain (MVA13) by overexpression of PspB (pAJM1), PspC (pAJM2) or PspBC (pAJM3) (using 0.02 % Ara). (b) Overexpression of PspC decreases pmf while co-expression with PspB counteracts this effect. Δ*ψ* was determined in a Δ*pspF* strain (MG1655Δ*pspF*) overexpressing PspB, PspC or PspBC. (c) Overexpression of PspBC directly induces *psp*. PspBC (pAJM3) or PspB^LeuZm^C (pGJ49; PspB^LeuZm^ does not transduce the *psp*-inducing signal) were co-expressed in either Δ*pspBC* (MVA45) or Δ*pspBC*Δ*arcB* (MVA83; Δ*arcB* diminishes *psp* induction) cells (using 0.02 % Ara). PspBC were co-expressed in a Δ*pspBC* strain (since the *arcB* mutation reduces induction by pIV; using 0.02 % Ara) in the presence of pIV. Vector, pBAD18-cm. As a control, *psp* expression was determined in the absence of PspA (Δ*pspA*, MVA27; to prevent negative regulation).

It is formally possible that the PspBC–PspA interactions that occur under non-stress conditions contribute to a steady-state level of a Psp[F–B]–ArcB regulatory complex. Alternatively, since the absence of PspBC does not release the negative regulation, and so cannot be required for formation of the inhibitory complex (Jovanovic *et al.*, 2009; Gueguen *et al.*, 2009), Psp[F–A] and Psp[C–B]–ArcB subcomplexes may communicate only in the presence of stress. Nevertheless, in both cases the stress-induced activation of *psp* may follow conformational changes in PspB and PspC that lead to release of negative regulation of PspA, presumably through altered protein–protein interactions. Interestingly, overexpression of PspC or PspBC, but not PspB alone, induces *psp* (Weiner *et al.*, 1991; Maxson & Darwin, 2006) (also see Fig. 5a and Supplementary Fig. S1A). Since overexpression of PspC decreases pmf in Δ*pspF* cells (i.e. where the Psp response is absent) (Fig. 5b) it may, similarly to pIV (Jovanovic *et al.*, 2006, 2009) or PulD (Guilvout *et al.*, 2006), act as an inducing stress for *psp* (Fig. 5a). In contrast, overexpression of PspBC strongly induces *psp* (Fig. 5a), but does not reduce pmf (Fig. 5b). Apparently, PspB (in the context of the PspBC complex) appears to prevent PspC from changing pmf, consistent with the observation that the growth rate in exponential phase is normal in Δ*pspC* cells overexpressing PspBC but severely impaired (10-fold) in Δ*pspC* cells overexpressing PspC alone (data not shown). Since PspB counteracts the ‘toxic’ effect of PspC, the PspBC proteins may be acting as an antitoxin–toxin pair as suggested by Brown & Shaw (2003). A parallel study (Gueguen *et al.*, 2009) showed that in *Y. enterocolitica* PspB stabilizes expression of WT PspC, a result differing from our *E. coli* data (Supplementary Fig. S1A).

Notably, overexpression of PspBC results in higher *psp* induction than does overexpression of PspC alone (Fig. 5a) (consistent with Weiner *et al.*, 1991 and Maxson & Darwin, 2006) – at a level comparable to uncontrolled *psp* expression in Δ*pspA* cells (Fig. 5c). In line with this observation, co-expression of PspBC in the presence of pIV did not result in increased *psp* induction (Fig. 5c and Supplementary Fig. S1B). Since PspBC overexpression does not simply induce *psp* by decreasing pmf (i.e. the overproduced proteins do not in themselves generate a ‘stress’ signal) we suggest that overproduced PspBC resembles the ‘stressed on-state’ of the Psp response.

The effect of overproduced PspBC could directly alter the state of PspA, and hence negative regulation, thereby bypassing upstream signalling systems (e.g. the ArcAB system). To test this directly, we used a leucine zipper motif (LeuZ) mutant (LeuZm) of PspB (PspB^LeuZm^) that maintains the ability to interact with PspC but is no longer able to interact with ArcB and is therefore inactive in upstream signalling (Jovanovic *et al.*, 2009). When co-expressed with PspC, this mutant form of PspB should support *psp* induction independent of any upstream signalling event. Significantly, overexpression of PspB^LeuZm^C resulted in a similar level of *psp* induction as overexpressed WT PspBC (Fig. 5c). In line with this, overexpression of either PspBC or PspB^LeuZm^C strongly induces *psp* in the absence of ArcB (Δ*arcB* strain) (Fig. 5c). These data argue that a PspBC complex is sufficient to induce the Psp response, by relieving PspA-imposed inhibition, in the absence of ArcB.

### PspC residues 40–119 are required for *psp* induction

**Fig. 6.**
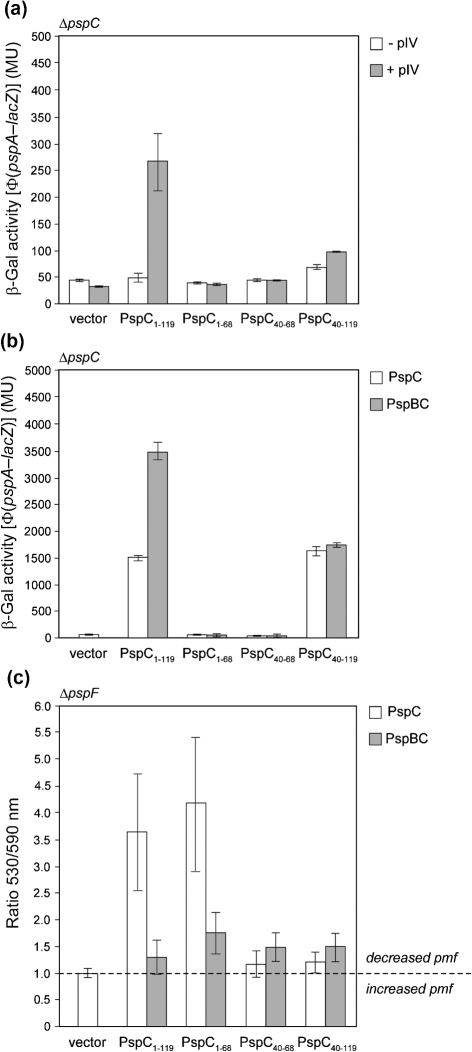
*psp* expression by the PspC fragments. (a) Full-length PspC is required for signal transduction upon pIV-dependent *psp*-inducing stress. Induction of the chromosomal Φ(*pspA–lacZ*) fusion in a Δ*pspC* strain (MVA13) expressing a low level of PspC (1–119, pAJM2) or PspC fragments (1–68, pAJM7; 40–68, pAJM5; 40–119, pAJM8) (using 0.001 % Ara) in the absence or presence of pIV (pGJ4) (see Methods). (b) The TM-periplasmic region of PspC (PspC_40–119_) is sufficient for PspB-independent induction of *psp*. Induction of the chromosomal Φ(*pspA–lacZ*) fusion in a Δ*pspC* strain (MVA13) overexpressing PspC (1–119, pAJM2) or PspC fragments on its own (as in a) or with PspB [PspBC (pAJM3) or PspBC fragments (1–68, pAJM12; 40–68, pAJM10; 40–119, pAJM13)] (using 0.02 % Ara) (see Methods). (c) Overexpression of PspC_1–68_ decreases pmf while co-expression with PspB counteracts this effect in a Δ*pspF* strain (MG1655Δ*pspF*).

We next addressed which determinants were important for PspBC–PspA signalling. Recall that PspA–PspC and PspB–PspC, but not PspB–PspA, were shown to directly interact (see above), suggesting that PspC may have a central role in transducing the stress signal to PspA. We first truncated PspC based on its predicted domain structure and topology (Fig. 1) to form three PspC fragments: PspC_1–68_ (cytoplasmic and TM domain), PspC_40–68_ (TM domain) and PspC_40–119_ (TM-periplasmic domain). In the absence of PspC (Δ*pspC*), induction of the psp response upon pIV stress no longer occurs (reviewed by Darwin, 2005; see also Jovanovic *et al.*, 2009). We initially introduced into Δ*pspC* cells either full-length PspC (residues 1–119) or one of the three fragments to test their activity with regard to their ability to support *psp* induction upon pIV stress. Each protein was expressed at a level similar to the basal level that was insufficient to induce *psp* in the absence of stress, and was scored for supporting pIV-dependent induction of *psp* (Fig. 6a). Only full-length PspC complemented the Δ*pspC* mutation for *psp* induction by pIV. Notably, similar levels of pIV expression were detected in all the samples tested (Supplementary Fig. S2A), and expression of wild-type PspC was at least 20-fold lower than that following pIV induction of chromosomal *psp* (Supplementary Fig. S2B). These results establish that full-length PspC, but not its fragments, supports signal transduction to PspA in pIV-dependent induction of *psp*.

PspC and its fragments (tested separately or co-expressed with PspB) were strongly overexpressed in Δ*pspC* cells at a level that was sufficient for PspC or PspBC (see Fig. 5a) to induce *psp*. Under these conditions the PspC_40–119_ fragment induced *psp* expression even in the absence of co-expressed PspB (Fig. 6b). We do note that expression levels of the PspC fragments varied greatly (Supplementary Fig. S2C); however, these differences do not account for the inability of some fragments to induce *psp*. For example, the PspC_1–68_ and PspC_40–119_ fragments are expressed similarly in the absence of PspB (Supplementary Fig. S2Ci and Di), yet only the PspC_40–119_ fragment induces *psp* (Fig. 6b). Further, the fragments did not appear to mislocalize (Supplementary Fig. S2D). Notably, expression of the PspC_40–68_ and PspC_40–119_ fragments seemed to be stabilized in the presence of co-overexpressed PspB, potentially via interactions between the TM domains of these two proteins (see below). As an additional control, we determined Δ*ψ* in Δ*pspF* cells (where *psp*-inducing agents, such as pIV, decrease pmf in the absence of the Psp response) overexpressing the PspC fragments, including PspC_40–119_ (in either the absence or presence of PspB), and found that pmf was not decreased (Fig. 6c), establishing that PspC_40–119_ does not act as a typical *psp*-inducing stress agent. Importantly, overexpression of the PspC_1–68_ fragment in the absence of co-expressed PspB decreased pmf (Fig. 6c) and impaired cell growth in Δ*pspC* cells similarly to full-length PspC (data not shown), but was unable to induce *psp*. Therefore, even when pmf was reduced, the PspC_1–68_ fragment was unable to complement a Δ*pspC* mutation and support *psp* induction. These data suggest that the PspC_1–68_ sequence lacking the putative periplasmic domain is important for the ‘toxic’ effect of PspC (i.e. a drop in pmf, see above). The PspC putative periplasmic domain (residues 69–119, present in the PspC_40–119_ fragment that induces *psp* but not in the PspC_1–68_ or PspC_40–68_ fragments, which do not induce *psp*) can function independently of upstream signalling and so may be directly involved in the release of the PspA–PspF inhibitory complex and hence functions positively in *psp* induction.

### The integrity of the putative leucine zipper motif of PspC is required for *psp* induction

The fragmentation approach demonstrated that the putative periplasmic region of PspC may be involved in *psp* induction and hence presumably might receive the inducing signal or contact PspA in order to release the negative regulation that PspA imposes on PspF.

Initially we analysed the ability of each of the PspC fragments to interact with PspB and PspA. Significantly, we observed that all the PspC fragments were capable of interacting with PspB or PspB^LeuZm^, demonstrating that this interaction (and also PspB self-association) does not require the PspB LeuZ (Table 1 and data not shown). These data also suggest that the determinants in PspC required for the interaction with PspB lie within the putative TM domain (residues 40–68) (Table 1 and Supplementary Fig. S2C). Importantly, and in contrast to WT PspC, two of the three PspC fragments failed to interact with PspA; only the C-terminal PspC_40–119_ Cya fusions interacted with PspA (Table 1 and Supplementary Table S2). This is somewhat surprising given the proposed topology of PspC, where the C-terminus is facing the periplasm (Kleerebezem *et al.*, 1996), suggesting that in the absence of the predicted cytoplasmic domain, the PspC_40–119_ fragment may adopt another topology.

**Fig. 7.**
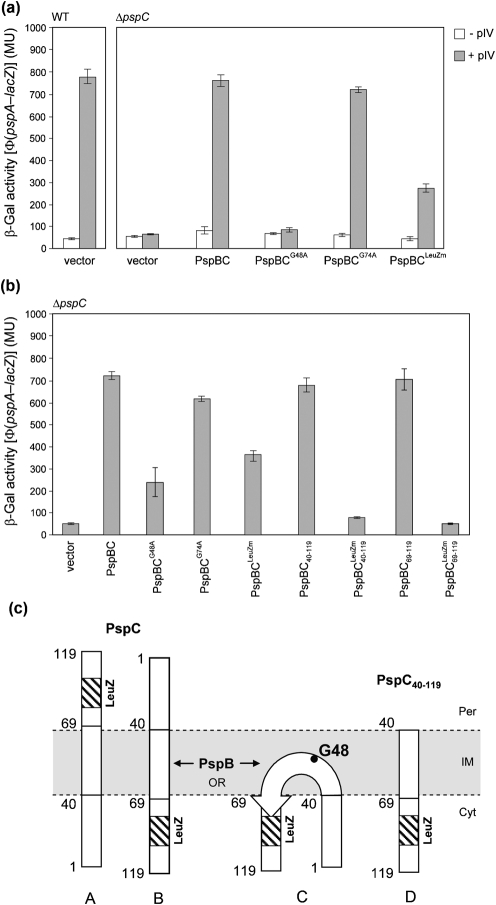
PspC determinants involved in signal transduction and induction of *psp*. (a) The PspC LeuZ and residue G48 are required for pIV-induced *psp* expression. Induction of the chromosomal Φ(*pspA*–*lacZ*) fusion in a Δ*pspC* strain (MVA13) expressing low-level PspBC (pAJM3) or PspBC mutants (PspC mutants: G48A, pGJ54; G74A, pGJ55; LeuZm, pGJ57) (using 0.001 % Ara) in the absence or presence of pIV (pGJ4) (see Methods). As a control, pIV-dependent induction of *psp* in WT cells (MVA4) is presented. (b) High-level co-expression of PspBC^G48A^, PspBC^LeuZm^, 
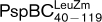
 (pGJ60) and 
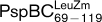
 (pGJ61) mutants failed to directly induce *psp* expression. Induction of the chromosomal Φ(*pspA–lacZ*) fusion in a Δ*pspC* strain (MVA13) by overexpression of PspBC or PspBC mutants (using 0.02 % Ara). (c) The PspC periplasmic region may exist in two topologies: schematic illustrating the potential topologies of PspC (A, B or C) and PspC_40–119_ (the periplasmic region containing the LeuZ; D).

The sequence assigned to the PspC periplasmic domain contains a putative LeuZ (Kleerebezem *et al.*, 1996) (Fig. 1b). When we examined the activities of LeuZ mutants (LeuZm) in the context of full-length PspC (PspC^LeuZm^) and the PspC_40–119_ fragment (
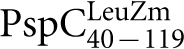
) (see Supplementary Table S1) we found that both proteins failed to interact with PspA (Table 1 and Supplementary Table S2) but maintained an ability to interact with PspB (Table 1). Given that the LeuZ of PspC appears important for PspA interactions, we reasoned that PspC proteins that contain a disrupted LeuZ would no longer be active for *psp* induction (assuming that they no longer relieve the inhibition imposed by PspA that is mediated by protein–protein interactions). Since full-length PspC is strictly required for *psp* induction upon pIV stress (Fig. 6a), and to avoid affecting pmf (Fig. 6c), the signal transduction complementation assays were performed in Δ*pspC* cells co-expressing PspB and full-length PspC (Fig. 7a). PspBC^LeuZm^ when expressed at a low level in the presence of pIV was unable to fully induce the *psp* response (Fig. 7a; compared to WT PspBC). The expression level of pIV under all conditions assayed was similar (Supplementary Fig. S3A), indicating that differences that arise in *psp* induction are unlikely to be due to variations in the level of the primary stress signal. The LeuZ of PspC appears to be important for signalling and/or the outcome of signalling to PspA.

When co-expressed with PspB, the PspC LeuZm in the context of either WT PspC (PspC^LeuZm^) or the PspC_40–119_ fragment (
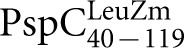
) was either able (to some extent) or unable, respectively, to induce *psp* (Fig. 7b). Significantly, the expression level and localization of PspC^LeuZm^ were similar to those of WT PspC (Supplementary Fig. S3B and C), further suggesting that a loss of productive interactions with PspA and the subsequent reduction in *psp* induction is not simply a consequence of protein misfolding or mislocalization.

Recent parallel studies (Gueguen *et al.*, 2009) demonstrated that in *Y. enterocolitica* the periplasmic region and LeuZ of PspC were important in *psp* induction. Gueguen *et al.* (2009) proposed a model in which the periplasmic region of PspC (more specifically the LeuZ) receives the stress signal rather than directly interacting with PspA. In addition, they showed that complementation of a chromosomal *pspC* deletion with PspC variants harbouring cytoplasmic domain truncations led to constitutive *psp* expression. These outcomes resemble the activities of the PspC_40–119_ fragment reported here. Gueguen *et al.* (2009) proposed that the cytoplasmic domains of PspC and PspB are involved in the negative regulation of *psp* in the absence of stress, and then interact with the Psp[A–F] inhibitory complex upon stress to release PspF negative regulation. However, Gueguen *et al.* (2009) found that the PspC cytoplasmic domain truncations still required the putative periplasmic domain to exhibit constitutive *psp* expression. Our results suggest that the PspC putative periplasmic domain may function in a direct interaction with PspA for release of negative regulation rather than just in receiving the inducing signal.

### Characterization of the PspC_69–119_ fragment

To further dissect the possibility that the putative PspC periplasmic domain (residues 69–119) directly interacts with PspA, we characterized the PspC_69–119_ fragment (see Supplementary Table S1), which contains the sequence predicted to reside in the periplasm. PspC_69–119_ expressed in the absence (Supplementary Fig. S2Ci) and presence (Supplementary Fig. S2Cii) of PspB was detected within the soluble cell fraction (Supplementary Fig. S2Dii). Overexpression of PspC_69–119_ does not significantly change Δ*ψ* (data not shown). Similarly to PspC_40–119_, overexpression of PspC_69–119_ in the absence of stress strongly induced *psp* but only when co-expressed with PspB (Fig. 7b). In addition, induction of *psp*, by overexpressed PspBC_40–119_ or PspBC_69–119_ is independent of ArcB (data not shown). These data clearly indicate that PspB, in concert with the periplasmic domain of PspC, directs the release of negative regulation of *psp* expression. The Cya–PspC_69–119_ fusion interacted with Cya–PspA (but not PspB–Cya) only when co-expressed with WT PspB (Table 1 and Supplementary Table S2). 
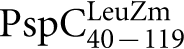
 (see Supplementary Table S1 and Supplementary Fig. S2Cii), when co-overexpressed with PspB, was unable to induce *psp* (Fig. 7b) or interact with PspA (Table 1 and Supplementary Table S2). These results are consistent with the previously demonstrated PspA–PspB interaction observed only in the presence of PspC (Adams *et al.*, 2003) and here we show that the PspC LeuZ is probably needed for this interaction. Taken together with results obtained with the PspC_40–119_ fragment (see above), direct PspC_69–119_ binding interactions with PspA seem to support induction of *psp*.

Our results show that the LeuZ located in the putative periplasmic domain of PspC is strictly required for PspA interactions and by clear inference *psp* induction – potentially explaining why PspC constitutive mutants still require the periplasmic domain for activation of *psp* expression (Gueguen *et al.*, 2009). The results obtained with the PspC_40–119_ and PspC_69–119_ fragments in terms of *psp* induction and the BACTH assays, and assuming the periplasmic domain of PspC should interact with the cytoplasmic face of PspA, suggest that the periplasmic domain of PspC would favour one topology when pIV is expressed in order to directly transduce the stress signal to the Psp[A–F] regulatory complex. A phenocopy of the stress-induced ‘topology switch’ may be simply realized by mass-action effects: in the absence of stress PspC may exist in equilibrium between active and inactive conformations potentially reflecting two distinct topologies of PspC (and so controlling basal level expression of *psp* genes); overproduction of PspC would result in more of the activating ‘on-state’ PspC topology being present in the cell, resulting in *psp* induction.

### PspC residue G48 is a determinant in *psp* induction

The possibility that the periplasmic domain of PspC may exist in two states (‘in’ and ‘out’), possibly altering its subcellular localization in response to stress, prompted us to apply the positive-inside rule (von Heijne, 1992) to the PspC sequence using different topology prediction programs (see Methods and Supplementary Fig. S4). The results were as follows: tmhmm – N-terminus inside, C-terminus outside; hmmtop – N-terminus outside, C-terminus inside; TopPred – N terminus outside, C-terminus inside; topcons – N-terminus inside, C-terminus inside with two potential TM domains and a small periplasmic loop (see Supplementary Fig. S4). These programs indicate that both topology scenarios (with respect to the periplasmic domain) are predicted for PspC, suggesting that the barrier between these two states is not high and that a topology switch potentially exists (Supplementary Fig. S4). In contrast, if we use the same programs with either PspB or PspG a single topology outcome is obtained, suggesting a relatively stable single topology state (data not shown). The localization of positive residues in extra-membrane domains is important for the positive-inside rule application and depends on Δ*ψ*, so protein topology can be dynamic (Bogdanov *et al.*, 2002, 2008; Zhang *et al.*, 2003, 2005). Potentially relevant for the Psp system and the PspBC sensors is that the observed reversible topological organization within some *E. coli* membrane proteins (e.g. LacY, PheP, GabP) is governed by a change in membrane phospholipid composition (reviewed by Bogdanov *et al.*, 2009; Dowhan & Bogdanov, 2009). Further, kinked hydrophobic TM helices (such as the ones obtained in the PspC topology predictions; Supplementary Fig. S4), particularly those containing glycine (G) residues, are proposed to bend more easily within the membrane than those with straight TM helices (Jiang *et al.*, 2002). Notably, these glycine residues are also thought to mediate the protein–protein interactions in polytopic membrane proteins (Javadpour, *et al.*, 1999). PhoA fused to the PspC region predicted to reside in the periplasm showed activity (Kleerebezem *et al.*, 1996), but it has not been discounted that this region can also at times be localized in the cytoplasm (e.g. using a fusion to *β*-galactosidase).

In light of the data concerning polytopic membrane proteins, we analysed the sequence of the putative TM helix (residues 40–68) of PspC and located a highly conserved glycine residue at position 48. When we mutated this residue to alanine (PspC^G48A^) we found that, similar to PspC^LeuZm^, PspC^G48A^ failed to induce the *psp* response (Fig. 7a, b and Supplementary Fig. S3B, C). In agreement with these observations, the BACTH assay illustrates that PpsC^G48A^ is no longer able to interact with PspA (or, interestingly, PspG); however, its ability to interact with PspB remains unaffected (Table 1). As a control we constructed the PspC^G74A^ mutant, where residue G74 is predicted to lie within the periplasmic domain and as such should not affect the topology of the TM domain. As predicted, this mutant retained WT PspC activities in terms of signal transduction and *psp* induction (Fig. 7a, b and Supplementary Fig. S3B, C) and Psp protein interactions (Table 1). These results suggest that the highly conserved residue G48 may act as a determinant in regulating or establishing the activity of PspC in the stress signalling pathway. It is unlikely that G48 directly contacts PspA, given that this residue is predicted to lie within the IM and PspA is a peripheral IM protein. Rather, G48 might be required to establish the correct conformation and topology of PspC for an interaction with PspA.

Notably, the inability of PspC^G48A^ to interact with PspG (in contrast to the PspC^LeuZm^ variant) (Table 1) indicates that residue G48 may direct a specific interaction with PspG, which could have implications for the function of PspG under stress. Consistent with this view, the PspC_40−68_ fragment retains the ability to interact with PspG (Table 1), further suggesting that PspC IM interactions (with PspG) are critical for PspG functionality.

### Concluding remarks

The analyses presented here demonstrate that PspB and PspC interact within the IM and may well serve as a scaffold within the Psp regulatory complex to which ArcB, PspA and PspG bind. We show, for the first time, that both PspB and PspG have a clear tendency to self-associate – in contrast to PspC – and identify determinants within PspB and PspC required for regulatory complex formation and *psp* induction. Since the experiments were conducted in the presence of co-expressed PspBC (to prevent alterations in pmf, which could ultimately lead to *psp* induction), and within the signalling experiments the amount of PspBC production was not elevated above basal levels, we expect that the outcomes discussed here are physiologically relevant.

In particular, we have established that residues 40–119 of PspC, which include the putative LeuZ (in the periplasmic domain) and residue G48 (in the TM domain), are important determinants required for signal transduction during pIV-dependent *psp* induction and consequently appear to have important effects on whether PspC interacts with PspA (and PspG for G48). We suggest that PspC may function as a membrane protein with more than one topology, possibly dynamic protein topologies (Fig. 7c). PspC topologies may respond to changes in the physical/chemical properties of the IM and Δ*ψ*. Apparently the PspC_40–119_ fragment (which has no predicted cytoplasmic domain), which localizes in the IM, has the ability (according to the positive-inside rule) to position the periplasmic domain within the cytoplasm, and to induce *psp* in the absence of PspB (Fig. 7c). Moreover, the soluble PspC_69–119_ fragment (carrying only the periplasmic domain) in the presence of co-expressed PspB interacts with PspA and induces *psp*. Since neither interaction of PspB with PspC_69–119_ nor interaction of PspA with PspB was detected, PspA–PspC_69–119_–PspB interactions may be highly co-operative, with very weak initial single component interactions. The interaction with PspA and induction of *psp* is dependent on the PspC periplasmic domain LeuZ. Considering that PspB–PspC interactions are both PspC LeuZ- and PspB LeuZ-independent (see Table 1) and that PspC_40–119_ but not 
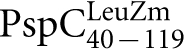
 can interact with PspA in the absence of PspB (see Table 1 and Supplementary Table S2), we argue against PspC working through PspB. Additionally, we show that one ‘toxic’ effect of PspC (the drop in pmf) is counteracted by PspB. This finding indicates that PspC and PspB may be acting as a ‘toxin–antitoxin’ pair that may help to stabilize PspC in a topology or ‘active on-state’ favourable for interacting with PspA (Fig. 7c).

Finally, considering a potential mechanism of *psp* induction involving two distinct PspC topologies, we suggest that it is formally possible that another signal specifically recognized by PspC (or PspBC) may involve changes in the physical/chemical properties of the membrane, given that inhibitors of phospholipid biosynthesis (Bergler *et al.*, 1994) or application of free fatty acids (Weiner & Model, 1994) strongly induce the *psp* response. In addition, transcription profiling revealed a link between PspA and use of glycerol 3-phosphate in the biosynthesis of phospholipids (Jovanovic *et al.*, 2006). Further, PspA was shown to bind specific IM phospholipids, phosphatidylglycerol and phosphatidylserine to exhibit its effector function (Kobayashi *et al.*, 2007). In agreement, outer-membrane secretins mislocalized within the IM or a block in protein translocation were shown to induce *psp* (reviewed by Model *et al.*, 1997; Darwin, 2005). Negatively charged phospholipids were found to restore translocation of outer-membrane precursor proteins across the phosphatidyglycerol-depleted IM of *E. coli* cells (Kusters *et al.*, 1991). Notably, archaea and acidophilic bacteria that have an inverted Δ*ψ* and different membrane content have a Psp system that contains only the PspA homologue and no recognizable PspBC proteins (Bidle *et al.*, 2008; Vrancken *et al.*, 2008), perhaps because PspBC function with a particular membrane potential and membrane lipid composition.

## Figures and Tables

**Table 1.** Interactions between the Psp proteins *in vivo* The BACTH system was used to detect protein–protein interactions between the Psp and ArcB proteins *in vivo*. Negative control, BTH101/pKT25+pUT18C vectors alone (85±9 MU); positive control, BTH101/pKT25-zip+pUT18C-zip (1405±64 MU); N-terminal (‘n’) protein fusions with T25 or T18 Cya fragments, respectively; C-terminal (‘c’) protein fusions with T25 or T18, respectively. Interaction estimates were as follows: +/−, weak interaction (283–470 MU); +, interaction (471–940 MU); ++, strong interaction (>940 MU); −, no interaction (≤282 MU); nd, not determined. See Methods for construction of fusion proteins, growth conditions and calculation of mean values.

**Protein fusion**	**T25–‘n’**							**‘c’–T25**	
	**PspA**	**PspC**	**PspC_1–68_**	**PspC_40–68_**	**PspC_40–119_**	**PspC_69–119_**	PspC_69–119_+PspB*	**PspB**	**PspG**
**T18–‘n’**									
PspA	+	+†	−	−	−	−	+	−	−
PspC	+	−†‡	−	−	−	nd	nd	++‡	+
PspC_1–68_	−	−	−	−	−	nd	nd	++	nd
PspC_40–68_	nd	−	−	−	−	nd	nd	+	+
PspC_40–119_	−	−	−	−	−	nd	nd	+	nd
PspC^LeuZm^	−	nd	nd	nd	nd	nd	nd	+	+/−
PspC_69–119_	−	nd	nd	nd	nd	nd	nd	−	nd
PspC_69–119_+PspB*	+	nd	nd	nd	nd	nd	nd	nd	nd
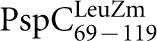 +PspB*	−	nd	nd	nd	nd	nd	nd	nd	nd
PspC^G48A^	−	nd	nd	nd	nd	nd	nd	++	−
PspC^G74A^	+	nd	nd	nd	nd	nd	nd	++	+
ArcB	+/−	+/−†	nd	nd	nd	nd	nd	+/−	−
ArcB*	+/−	+/−†	nd	nd	nd	nd	nd	+/−	−
ArcB^LeuZm^	+/−	+/−†	nd	nd	nd	nd	nd	+/−	−
**‘c’–T18**									
PspC_40–119_	+	nd	nd	nd	nd	nd	nd	+	nd
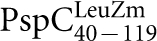	−	nd	nd	nd	nd	nd	nd	+	nd
PspB	−	++†	++	++	++	−	nd	++	−
PspB^LeuZm^	−	++†	++	++	++	nd	nd	++	nd
PspG	−	+	nd	nd	nd	nd	nd	−	++

*PspB co-expressed from plasmid pAJM1; experimental results already presented elsewhere confirmed in this work and used as controls. †Jovanovic *et al.* (2009). ‡Maxson & Darwin (2006).

## Abbreviations

- Δ*ψ*, electron potential
- Ara, l-arabinose
- BACTH, bacterial two-hybrid
- *β*-Gal, *β*-galactosidase
- IM, inner membrane
- LeuZ, leucine zipper motif
- LeuZm, leucine zipper mutant
- pIV, protein IV
- pmf, proton-motive force
- Psp, phage-shock protein
- Sol, soluble fraction
- MU, Miller units
- TM, transmembrane
